# Magnetic Resonance Elastography of Anterior Mediastinal Tumors

**DOI:** 10.1002/jmri.29481

**Published:** 2024-06-10

**Authors:** Lina Zhou, Qin Peng, Wei Tang, Ning Wu, Lin Yang, Linlin Qi, Jiang Li, Yao Huang

**Affiliations:** ^1^ Department of Diagnostic Radiology National Cancer Center/National Clinical Research Center for Cancer/Cancer Hospital, Chinese Academy of Medical Sciences and Peking Union Medical College Beijing China; ^2^ Department of Radiology Fudan University Shanghai Cancer Center Shanghai China; ^3^ Department of Oncology Shanghai Medical College, Fudan University Shanghai China; ^4^ Department of Pathology National Cancer Center/National Clinical Research Center for Cancer/Cancer Hospital, Chinese Academy of Medical Sciences and Peking Union Medical College Beijing China; ^5^ Office for Cancer Screening, National Cancer Center/National Clinical Research Center for Cancer/Cancer Hospital, Chinese Academy of Medical Sciences and Peking Union Medical College Beijing China

**Keywords:** mediastinal neoplasms, elasticity imaging techniques, lymphoma, thymus neoplasms

## Abstract

**Background:**

Preoperative differentiation of the types of mediastinal tumors is essential. Magnetic resonance (MR) elastography potentially provides a noninvasive method to assess the classification of mediastinal tumor subtypes.

**Purpose:**

To evaluate the use of MR elastography in anterior mediastinal masses and to characterize the mechanical properties of tumors of different subtypes.

**Study Type:**

Prospective.

**Subjects:**

189 patients with anterior mediastinal tumors (AMTs) confirmed by histopathology (62 thymomas, 53 thymic carcinomas, 57 lymphomas, and 17 germ cell tumors).

**Field Strength/Sequence:**

A gradient echo‐based 2D MR elastography sequence and a diffusion‐weighted imaging (DWI) sequence at 3.0 T.

**Assessment:**

Stiffness and apparent diffusion coefficients (ADC) were measured in AMTs using MR elastography‐derived elastograms and DWI‐derived ADC maps, respectively. The aim of this study is to identify whether MR elastography can differentiate between the histological subtypes of ATMs.

**Statistical Tests:**

One‐way analysis of variance (ANOVA), two‐way ANOVA, Pearson's linear correlation coefficient (*r*), receiver operating characteristic (ROC) curve analysis; *P* < 0.05 was considered significant.

**Results:**

Lymphomas had significantly lower stiffness than other AMTs (4.0 ± 0.63 kPa vs. 4.8 ± 1.39 kPa). The mean stiffness of thymic carcinomas was significantly higher than that of other AMTs (5.6 ± 1.41 kPa vs. 4.2 ± 0.94 kPa). Using a cutoff value of 5.0 kPa, ROC analysis showed that lymphomas could be differentiated from other AMTs with an accuracy of 59%, sensitivity of 97%, and specificity of 38%. Using a cutoff value of 5.1 kPa, thymic carcinomas could be differentiated from other AMTs with an accuracy of 84%, sensitivity of 67%, and specificity of 90%. However, there was an overlap in the stiffness values of individual thymomas (4.2 ± 0.71; 3.9–4.5), thymic carcinomas (5.6 ± 1.41; 5.0–6.1), lymphomas (4.0 ± 0.63; 3.8–4.2), and germ cell tumors (4.5 ± 1.79; 3.3–5.6).

**Data Conclusion:**

MR elastography‐derived stiffness may be used to evaluate AMTs of various histologies.

**Level of Evidence:**

4.

**Technical Efficacy:**

Stage 2.

Primary anterior mediastinal tumors (AMTs) are a heterogeneous group of neoplasms that can develop from several cell lineages.^1^ These tumors account for 50% of all mediastinal masses.^2^ Imaging assessment and accurate histopathological information play essential roles in tumor identification, staging, and follow‐up monitoring for recurrence.^3^

When an AMT is detected, chest radiography, ultrasonography, computed tomography (CT), magnetic resonance imaging (MRI), or positron emission tomography can be used for imaging assessment.^4^ Specific demographic characteristics, combined with clinical evidence and imaging features, may be used to determine the etiologies in most patients confidently.^5^, ^6^ However, some overlap has been found in morphological features, and additional information may be required to distinguish between different tumor types, narrow the differential diagnosis, and guide further management.^7^ MRI has advantages over routine CT in characterizing anterior mediastinal lesions in assessing anatomical extent and tissue composition.^8^, ^9^ With the advancement of diagnostic protocols over the last few decades, MRI techniques have become robust and versatile tools for the noninvasive assessment of cancer morphology and functionality.^10^ Diffusion‐weighted imaging (DWI) quantifies the diffusion of water molecules in tissues.^11^ Increased cellularity and vascularity are hallmarks of cancer and restrict diffusion, resulting in lower apparent diffusion coefficient (ADC) values. Previous studies have suggested that DWI can be applied to characterize anterior mediastinal tumors, particularly in identifying cystic components^12^, ^13^ and distinguishing malignancies from benign tumors.^14^, ^15^, ^16^ However, inconsistent diagnostic accuracies have been reported.^17^ False‐positive results occur with abscess and infective processes, and false‐negative results occur with cystic and necrotic lesions and in well‐differentiated neoplasms.^18^

Magnetic resonance (MR) elastography is a noninvasive technique used for the quantitative assessment of the mechanical properties of tissues in vivo. It is performed using a vibration source to generate low‐frequency mechanical waves in tissues, imaging the propagating waves using a phase‐contrast technique, and then processing the wave information to generate quantitative images showing mechanical properties such as tissue stiffness.^19^, ^20^ Since its first description by Muthupillai et al^21^ MR elastography has been successfully applied to solid organs, such as the liver, breast, brain, and extremities^21^, ^22^, ^23^, ^24^ and to the lungs, which have inherently poor 1H MR signals,^25^ as the elastic properties of biological materials are of interest as a diagnostic metric.^26^ However, studies on the performance of MR elastography in AMTs are lacking.

Thus, this study aimed to investigate the feasibility of MR elastography in characterizing the mechanical properties of AMTs with different histopathological subtypes.

## Materials and Methods

### Subjects

This study complied with the principles of the Declaration of Helsinki and was approved by our institutional Clinical Research Ethical Committee. Written informed consent was obtained from all participants. Between July 2018 and June 2022, 215 patients with anterior mediastinal masses detected by contrast‐enhanced MRI were initially recruited for this study. MRI examinations were performed to evaluate and preoperatively stage these lesions, and the MR elastography sequence was used for clinical applications in conjunction with routine, conventional MRI. The following patients were included in this study: 1) patients suspected of having conditions of a) primary anterior mediastinal masses and b) recurrent tumors untreated before MRI examinations; 2) patients who underwent routine MRI, DWI, and MR elastography using the same scanner; and 3) patients with tumors that were planned to be histopathologically confirmed as primary anterior mediastinal masses by subsequent resection or biopsy. The following patients were excluded: 1) patients with typical benign imaging findings on CT, such as pure cystic lesions or triangular thymic lesions; 2) patients with lesions that were not visible on elastography; and 3) patients with contraindications to MRI, such as claustrophobia. A final study group of 189 patients was enrolled for retrospective data analysis, confirmed by histopathological findings.

### Imaging Acquisition

All MRI studies were performed using a 3.0 T whole‐body scanner (Discovery MR750, GE Healthcare, Waukesha, WI, USA) equipped with a 32‐channel coil. The routine mediastinal imaging protocol included the following sequences: axial PROPELLER T2‐weighted imaging (T2WI) with fat suppression (FS), axial fast spin echo T1‐weighted imaging (T1WI) with FS, DWI, axial dynamic 3D fat‐saturated spoiled gradient echo sequence/liver acquisition with volume acceleration (LAVA) before and after administration of contrast agent, and two‐dimensional (2D) axial fast spoiled gradient echo. Scanning parameters are listed in Table 1. T2WI/FS was obtained in the supine position. The acquisition was started from above the thoracic inlet (at the level of the first thoracic vertebra, first ribs, and manubrium) to the upper part of the kidney. The DWI sequence was respiratory‐gated, and a b value of 800 was used. ADC maps were automatically generated on a workstation using an algorithm implemented on the GE scanner. The scanning ranges of the T1WI/FS, DWI, and LAVA sequences were 2 cm above and below the target lesions.

**TABLE 1:** Imaging Parameters of the MRI Sequences

Sequence	Repetition time (msec)	Echo time (msec)	SENSE factor	Flip angle (°)	Matrix (number of slices)	Field of view (cm)	Slice thickness (mm)	Respiratory compensation	Duration
T2WI/FS	7200	112	2	90	512 × 512 (25–40)	32–44	5	Free‐breathing	2–3 minutes
T1WI/FS	245	1.72	2	70	512 × 512 (15–20)	32–44	6–8	Breath‐hold	21 sec
DWI	6000–8000	55.8–58	2	90	256 × 256 (15–28)	32–44	5	Free‐breathing	35–55 sec
LAVA	3.70	1.67	2	90	512 × 512 (1056)	32–44	2	Respiratory gating	7–9 minutes
MRE	600	58.6	1 (CLEAR)	90	256 × 256 (6)	32–44	5	Breath‐hold	19 sec

T2WI/FS = T2‐weighted imaging with fat suppression; T1WI/FS = T1‐weighted imaging with fat suppression; DWI = diffusion‐weighted imaging; LAVA = liver acquisition with volume acceleration；MRE = MR elastography.

MR elastography was performed using a commercially available system (MR Touch; GE Healthcare, Waukesha, WI, USA) with a 19‐cm‐diameter, 1.0‐cm‐thick cylindrical passive driver that was placed against the center of the chest (sternum) adjacent to the anterior mediastinal mass, as localized on the baseline MR images. Continuous vibrations of 60 Hz were used in this study because higher‐frequency vibrations are attenuated and lower‐frequency vibrations have a low‐spatial stiffness resolution.^25^ During suspended respiration, a gradient echo‐based 2D MR elastography sequence was used to acquire wave images (Table 1). After acquisition, the MRI system automatically processed the wave image data to generate elastograms. The images were matched with the corresponding DWI images for qualitative analysis.

### Image Analysis

Two radiologists (Y.H. and W.T., with 30 and 15 years of experience in thoracic radiology, respectively) interpreted MRI, DWI, and MR elastography datasets. To assess the feasibility of the MR elastography technique in depicting elastic features of anterior mediastinal masses, the imaging quality of the elastograms was evaluated as adequate or inadequate by the two readers before they performed elasticity analysis. The two independent readers were blinded. After the two readers independently delineated the ROI, the third senior chief physician (35 years of experience) independently reviewed the elastography's quality to confirm the elastography's quality and re‐read the tumor scope. There was no difference in the ROI delineated by the two independent readers, and finally, the data were output and recorded by each radiologist. Inadequate MR elastography examinations were mainly caused by technical failure and included those where 1) wave images showed no wave propagation or 2) the anterior mediastinal mass was not visualized on the elastograms owing to interference fringes or substantial signal loss.

The first 20 patients enrolled sequentially, and the ROI delineated by two independent readers. The intraobservers ICCs for size, ADC, and stiffness were 0.815/0.787, 0.852/0.878, and 0.878/0.908, respectively. The results showed good self‐repeatability. Thus, the same reader analyzed the remaining cases once independently, and the third senior doctor confirmed the final results. Both readers reviewed all MR scans to measure the lesion size. Size was defined as the maximal transverse diameter of the tumor measured on axial T2WIs. The readers then evaluated the ADC and stiffness values using DWI and elastography. In each patient, a circular or elliptical region of interest (ROI; Fig. 2) covering >75% of the area of the mass was placed on the T2WI showing the maximal axial diameter of the tumor, avoiding the cystic components, if any. An identical ROI was positioned in the corresponding elastography slice (Fig. 2) and the mean stiffness (in kPa) was measured automatically. Mean ADC values (10^−3^ mm^2^/sec) in the same ROI were also quantified from the ADC maps.

### Histological Reference

One hundred and six patients underwent surgical resections of the lesions, and 20 patients underwent thoracoscopic surgeries. CT‐guided transthoracic core needle biopsies of the anterior mediastinal masses were performed in 63 patients, with samples preserved in 10% formalin and subjected to histological evaluations for diagnosis. In 98 cases of surgically resected thymomas and thymic carcinomas (61 thymomas and 37 thymic carcinomas, respectively), the National Comprehensive Cancer Network Clinical Practice Guidelines^27^ for thymomas and thymic carcinomas were used to determine whether the capsule had been invaded. All results were collected and reviewed with the MR elastography and DWI data.

### Statistical Analysis

Mean (± standard deviation) values with 95% confidence intervals (CI) were generated for tumor size, ADC, and stiffness values. Inter‐reader and intrareader reproducibility was evaluated using the intraclass correlation coefficient (ICC) with 95% CI. An ICC of >0.75 was considered indicative of good agreement.^28^ A *t*‐test for equal or unequal variances (Welch's *t*‐test) was performed after the equality of variance hypothesis was tested using the *F*‐test to test between‐group differences. A one‐way analysis of variance (ANOVA) was performed to test between‐subgroup differences, followed by the Newman–Keuls multiple comparisons test after confirming the equality of variance using the Levene test. ANOVA was performed to examine the differences in stiffness and ADC values between histological subtypes. Receiver operating characteristic (ROC) curve analysis was performed, and the area under the curve (AUC) used to determine the accuracy of MR elastography and ADC in differentiating lesions of different histopathologic subtypes. Sensitivity, specificity, positive predictive value (PPV), and negative predictive value (NPV) were calculated using optimal cutoff values. Statistical analyses were performed using SPSS (version 20.0, Chicago, IL, USA) for Windows. *P*‐values of <0.05 were considered statistically significant.

## Results

### Study Population

**FIGURE 1:**
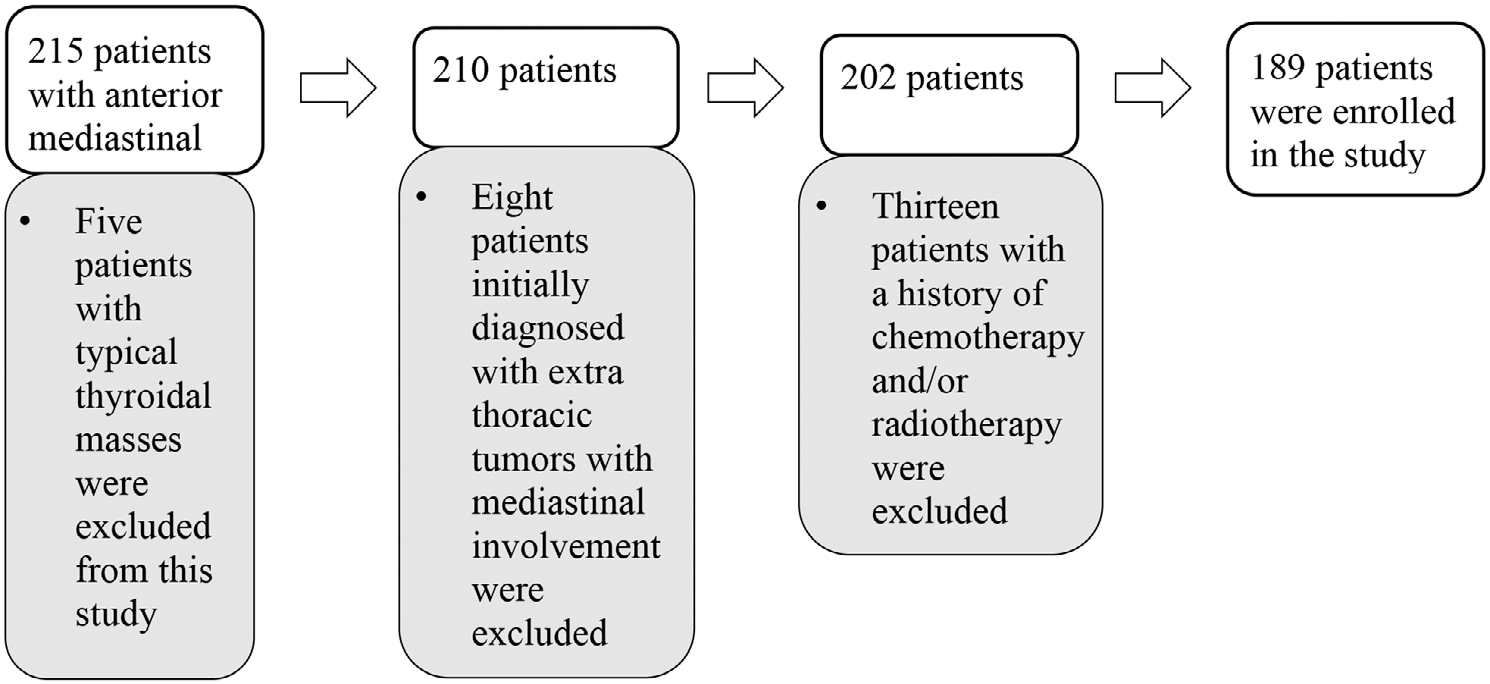
Flow chart summarizing patient selection.

One hundred and eighty‐nine patients (107 males and 82 females), including 62 with thymomas, 53 with thymic carcinomas, 57 with lymphomas (34 Hodgkin lymphomas and 23 non‐Hodgkin lymphomas), and 17 with germ cell tumors were included in this study. The study flowchart is illustrated in Fig. 1. The participant characteristics are listed in Table 2.

**TABLE 2:** Summary of General Characteristics and MR Imaging Findings According to Groups

Characteristic (Mean ± SD)	Lymphoma (*n* = 57)	Thymoma (*n* = 62)	Thymic Carcinoma (*n* = 53)	Germ Cell Tumors (*n* = 17)	*P*‐Value
Age (years)	33.3 ± 10.3	54.1 ± 11.5	53.1 ± 12.8	34.7 ± 13.0	–
Gender (Male/Female)	30/27	24/38	41/12	12/5	–
Size (cm)	6.8 ± 1.6	3.7 ± 1.8	6.5 ± 2.3	4.9 ± 2.2	0.06
ADC value (×10^−3^ mm^2^/sec)	1.51 ± 0.44	1.49 ± 0.51	1.46 ± 0.47	1.93 ± 0.72	0.17
Mean stiffness value (kPa)	4.0 ± 0.63	4.2 ± 0.71	5.6 ± 1.41	4.5 ± 1.79	≤0.01

SD = standard deviation; ADC = apparent diffusion coefficient.

### Image Quality Assessment and Interobserver Agreement

**FIGURE 2:**
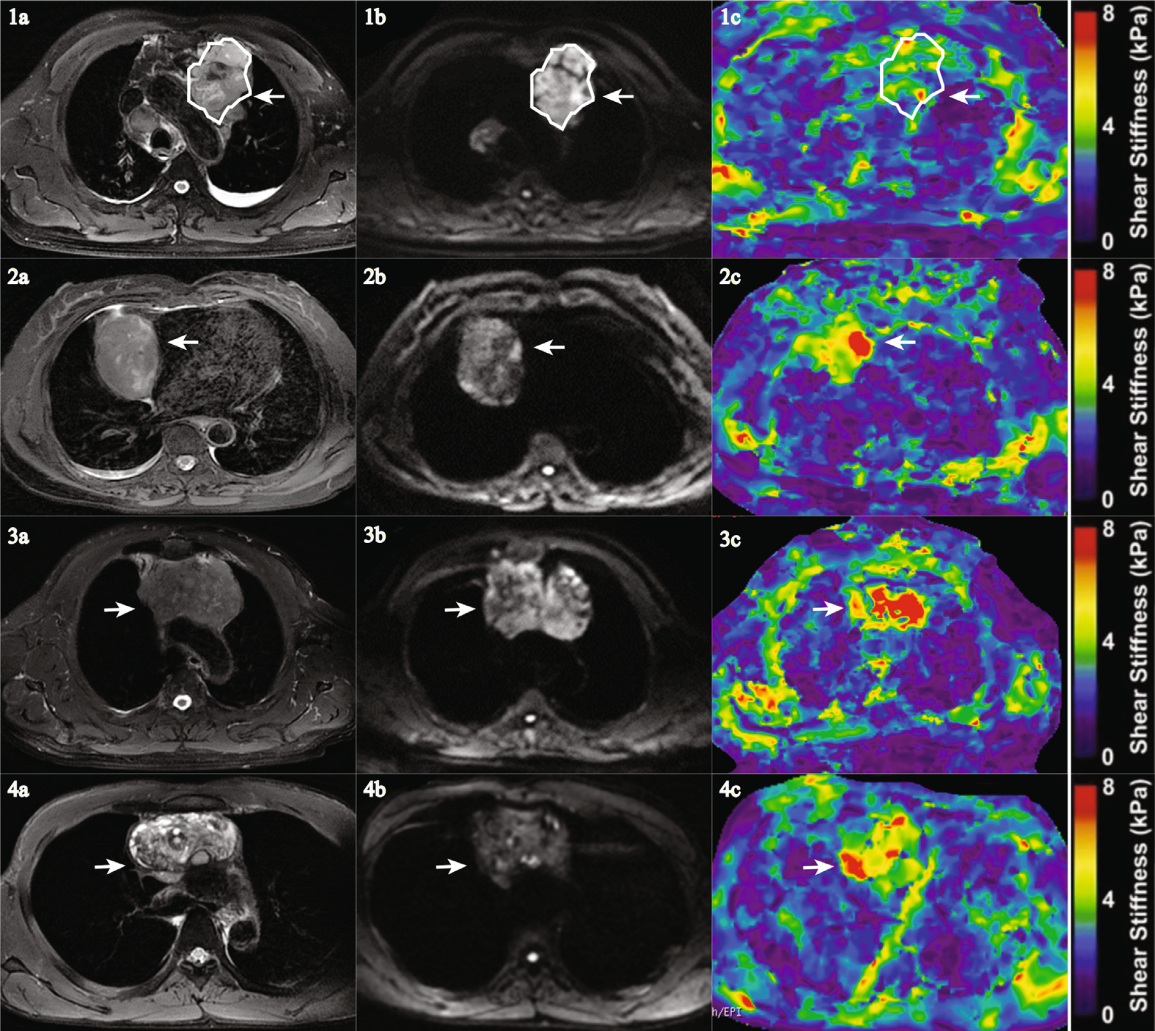
Typical MR elastography (MRE) cases of patients with different histopathological anterior mediastinum tumors (AMTs). An axial T2WI shows a mass (white arrow, 1a, 2a, 3a, and 4a) in the anterior mediastinum. A ROI (white line area) is placed within the mass on a DWI (1b) and copied to the corresponding slice of the elastogram (1c), where the shear stiffness is measured. For a 46‐year‐old female with pathologically proven lymphoma (1a–1c), the mean stiffness value was 3.9 ± 0.7 kPa. For a 67‐year‐old male with histopathologically proven thymoma (2a–2c, white arrow), the mean stiffness of the tumor was mean 4.6 ± 1.0 kPa. For a 60‐year‐old male with pathologically diagnosed thymic carcinoma (3a–3c, white arrow), the stiffness value of the tumor was 9.1 ± 1.4 kPa. For a 36‐year‐old male diagnosed with germ cell tumor (seminoma (4a–4c, white arrow)), the mean stiffness of the tumor was 4.5 ± 0.9 kPa. MRE = magnetic resonance elastography; AMTs = anterior mediastinal tumors; T2WI = T2‐weighted imaging; ROI = region of interest; DWI = diffusion‐weighted image.

The image quality of all elastograms and DWI images were adequate for all 189 patients. The inter‐observer ICCs calculated based on the two reader measurements for size, ADC, and stiffness were 0.786, 0.885, 0.966, respectively. The third senior chief physician (35 years of experience) independently reviewed the elastography's quality to confirm the elastography's quality and re‐read the tumor scope. There was no difference in the ROI delineated by the two independent readers. Therefore, all results were based on the first reader measurements. Images of representative cases are shown in Fig. 2.

### Comparison of MR Elastography and DWI Parameters and Diagnostic Approaches for the Distinction of Different Masses in the Anterior Mediastinum

**FIGURE 3:**
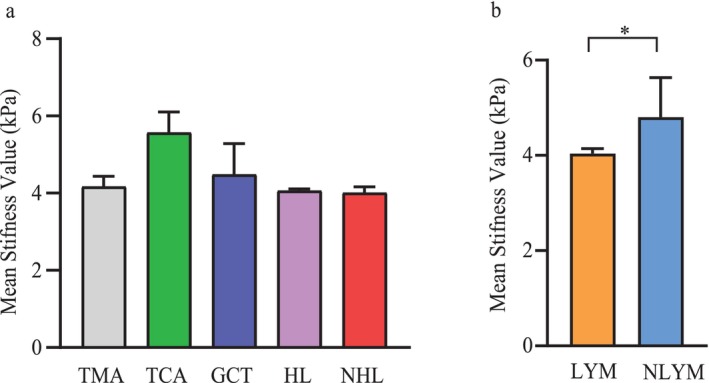
Bar whisker plot of different pathological subtypes of anterior mediastinal tumors. TMA = thymomas; TCA = thymic carcinomas; GCT = germ cell tumors; HL = Hodgkin lymphomas; NHL = non‐Hodgkin lymphomas; LYM = lymphomas; NLYM = other tumors in the anterior mediastinum that are not lymphoma; * *P* < 0.05.

Mean stiffness values were 5.6 ± 1.41 kPa for thymic carcinomas, 4.2 ± 0.71 kPa for thymomas, 4.0 ± 0.63 kPa for lymphomas, and 4.5 ± 1.79 kPa for germ cell tumors. Mean ADC values were 1.46 ± 0.47 × 10^−3^ mm^2^/sec for thymic carcinomas, 1.49 ± 0.51 × 10^−3^ mm^2^/sec for thymomas, 1.51 ± 0.44 × 10^−3^ mm^2^/sec for lymphomas, and 1.93 ± 0.72 × 10^−3^ mm^2^/sec for germ cell tumors (Fig. 3a). There was no significant difference in mean stiffness between thymomas and thymic carcinomas (*P* = 0.593).

The mean stiffness value of lymphomas was significantly lower than that of other tumors in the anterior mediastinum (4.0 ± 0.63 kPa vs. 4.8 ± 1.39 kPa, Fig. 3b). However, there was no significant difference in the ADC values between these two groups (1.51 ± 0.44 × 10^−3^ mm^2^/sec vs. 1.55 ± 0.54 × 10^−3^ mm^2^/sec, *P* = 0.305). Additionally, there was no significant difference in the mean stiffnesses between Hodgkin lymphomas and non‐Hodgkin lymphomas (4.1 ± 0.51 kPa vs. 4.0 ± 1.24 kPa, *P* = 0.400). There were also no significant differences in the stiffness values between lymphomas and thymomas (*P* = 0.432), or between lymphomas and germ cell tumors (*P* = 0.197).

The mean stiffness value of thymic carcinomas was significantly higher than that of the other tumors in the anterior mediastinum (5.6 ± 1.41 kPa vs. 4.2 ± 0.94 kPa). However, there was no significant difference in ADC values between these two groups (1.46 ± 0.47 × 10^−3^ mm^2^/sec vs. 1.57 ± 0.53 × 10^−3^ mm^2^/sec, *P* = 0.469). In addition, there were significant differences in the stiffness values between thymic carcinomas and lymphomas, between thymic carcinomas and thymomas, and between thymic carcinomas and germ cell tumors.

ROC curve analysis showed that the cutoff point of stiffness for optimal differentiation of lymphomas from other AMTs was 5.0 kPa (accuracy: 59%, sensitivity: 97%, specificity: 38%, PPV: 45%, NPV: 96%, AUC: 0.662, 95% CI: 0.556–0.767). ROC curve analysis also showed that the cutoff point of stiffness for optimal differentiation of thymic carcinomas from other AMTs was 5.1 kPa (accuracy: 84%, sensitivity: 67%, specificity: 90%, PPV: 72%, NPV: 88%, AUC: 0.791, 95% CI: 0.673–0.909).

## Discussion

The use of MR elastography to evaluate the lungs is technically challenging because of low‐proton density and susceptibility effects; however, spin‐echo‐based MR elastography sequences have shown promise for overcoming this limitation.^29^ Studies on the potential value of MR elastography in evaluating anterior mediastinal masses are lacking. The results of the current study provide preliminary evidence of the technical feasibility of characterizing the mechanical properties of masses in the anterior mediastinum.

### MR Elastography Assessment of the Stiffness of AMTs

This study showed that the mean stiffness of lymphomas was significantly lower than that of other tumor types in the anterior mediastinum. However, there was no significant difference in the ADC values. Lymphomas being “soft” may be due to their high cellularity, nuclear‐to‐cytoplasm ratio, and very little fibrous tissue or other extracellular matrix (ECM).^30^ It is known that the mechanical response of the ECM itself can play an important role in tumor formation.^31^, ^32^ Several factors may contribute to the increase in stiffness of other tumors as histopathologic processes, such as cell proliferation, angiogenesis, fibrosis, calcification, and necrosis, may cause changes in the viscoelastic properties of tissues.^33^, ^34^ Malignant tumors, such as thymic carcinomas, have abundant ECM and increased vascularity and interstitial pressure,^35^ which may result in increased stiffness. Thus, in this study, the mean stiffness of thymic carcinomas was also higher than that of other tumors in the anterior mediastinum. Lymphoma is a nonsurgical tumor, and radiotherapy and/or chemotherapy are the main therapeutic options.^36^, ^37^ MR elastography may help to distinguish lymphomas from other mediastinal tumors and avoid unnecessary surgery, which is difficult with conventional imaging techniques, such as CT and MRI.^38^

### Limitations

Considering the study was a relatively small number of cases, selection bias and potential bias are possible as magnetic resonance elastography (MRE) was performed in only a fraction of the cases.^3^ Since MR elastography has not been routinely used in the preoperative evaluation of mediastinal tumors at present, the patients enrolled in this study had tumors that were clearly shown on preoperative MRI. Therefore, the tumors included in this study were relatively large. The scope was clear, which may be one of the limitations of this exploratory study, and further exploration of the differential value of tumors with relatively small volumes are needed in the future. In the exploratory study, the senior readers were preferentially selected In another ongoing study of MRI, we will focus on the deviations in tumor ROI delineation by doctors with varying degrees of experience and on the analysis of the intra‐observer and inter‐observer repeatability differences among these doctors. The most important limitation of this study is its use of 2D MR elastography, which is currently the standard commercially available version of the technique. In 2D MR elastography, it is assumed that mechanical waves propagate along directions parallel to the plane of the section. If this condition is not satisfied, the calculated stiffness values will be less accurate. A 3D MR elastography may address this limitation.^39^

### Conclusion

This study has shown that MR elastography assessment of the mechanical properties of AMTs are promising for distinguishing histopathology, particularly lymphomas from other tumors in the anterior mediastinum.

## Funding Information

This research was supported by the Chinese Academy of Medical Sciences Initiative for Innovative Medicine (2017‐I2M‐1‐005), National Key R&D Program of China (2017YFC1308700), and Beijing Hope Run Special Fund of Cancer Foundation of China (LC2021A25).
